# Epigenetic Regulation of the Hippocampus, with Special Reference to Radiation Exposure

**DOI:** 10.3390/ijms21249514

**Published:** 2020-12-14

**Authors:** Genevieve Saw, Feng Ru Tang

**Affiliations:** Singapore Nuclear Research and Safety Initiative, National University of Singapore, Singapore 138602, Singapore; genevievesaw@nus.edu.sg

**Keywords:** epigenetics, hippocampus, radiation, histones, DNA methylation, miRNAs, glia

## Abstract

The hippocampus is crucial in learning, memory and emotion processing, and is involved in the development of different neurological and neuropsychological disorders. Several epigenetic factors, including DNA methylation, histone modifications and non-coding RNAs, have been shown to regulate the development and function of the hippocampus, and the alteration of epigenetic regulation may play important roles in the development of neurocognitive and neurodegenerative diseases. This review summarizes the epigenetic modifications of various cell types and processes within the hippocampus and their resulting effects on cognition, memory and overall hippocampal function. In addition, the effects of exposure to radiation that may induce a myriad of epigenetic changes in the hippocampus are reviewed. By assessing and evaluating the current literature, we hope to prompt a more thorough understanding of the molecular mechanisms that underlie radiation-induced epigenetic changes, an area which can be further explored.

## 1. Introduction

The hippocampus is a structure in the brain within the medial temporal lobe [1] which plays an important role in the limbic system and is involved in learning, memory processing and emotions [2]. The three principle structures of the hippocampal formation (hippocampus, dentate gyrus and subiculum) are made up of glutamatergic principal neurons and inhibitory GABAergic neurons [3], and damage to the hippocampus has been shown to bring about amnesic effects in humans, alluding to its major role in memory formation and consolidation [4]. Both animal and clinical studies have clearly indicated that radiation exposure or radiotherapy induces hippocampal damage, resulting in the impairment of neurogenesis and cognition [5]. The production of reactive oxygen species (ROS), pro-inflammatory cytokines and chemokines, pro-apoptotic proteins, autophagosome markers, excitatory neurotransmitters and neurotrophic factors may be involved in hippocampal neuropathy and cognitive impairment [6,7]. Recent studies have suggested that epigenetic mechanisms are involved not only in normal hippocampal development and regulation [8,9,10,11,12,13,14], but also in radiation-induced hippocampal pathophysiological changes leading to the development of neurological and neuropsychological disorders [15]. In this paper, we briefly introduce major mechanisms of epigenetic regulation, followed by a review of the epigenetic regulation of hippocampal neurogenesis. Normal physiological and pathophysiological regulation of different hippocampal cells, such as principal cells, interneurons and glial cells, by epigenetic mechanisms, is also summarized. We then focus on how low or high doses of radiation may induce epigenetic changes in the hippocampus and propose future research directions. 

### 1.1. Epigenetics

Epigenetics is a term first coined by C. H. Waddington in 1942 [16] without it being fully understood. It was said to refer to the interactions between genes and their surroundings [17] involving heritable changes in gene expression bringing about phenotypical changes without modifying the underlying DNA sequence [18]. Thus, the epigenome consists of covalent modifications of chromatin components, including DNA, RNA and histones, which ensure the inheritance of differentiated states [19]. Many studies now support the notion that epigenetic regulation plays significant and influential roles in biological systems throughout different stages of life, from development through to adulthood. Studies have shown that a reduction in cognitive development in early life correlated with differential methylation levels between hundreds of genes [20]. Due to the significance of its regulation, aberrant epigenetic regulation has been implicated in many diseases and disorders, such as in cancer, neurodegenerative diseases and congenital diseases [21,22,23,24,25]. There are three major mechanisms of epigenetic regulation, namely, DNA methylation, histone modifications and non-coding RNA targeting [26]. The dynamic interactions of chromatin modifications, cytosine modifications within DNA and miRNAs determine the complex outcomes of gene expression [11].

### 1.2. DNA Methylation

DNA methylation is a biological process in which methyl groups are added to the DNA, bringing about the conversion of cytosine to 5-methyl cytosine (5 mC) mediated by DNA methyltransferases (DNMTs), which then interferes with transcription initiation, leading to gene silencing [17]. DNA methylation plays an important role in epigenetic regulation, changes in chromatin structure, DNA repair and DNA–protein interactions [27]. CpG islands are DNA methylation regions in promoters that mediate gene expression regulation through transcriptional silencing [28].

The reversal of DNA methylation involves the removal of the methyl group from cytosines, converting 5 mC back to 5-hydroxymethlcytosine (5 hmC). DNA demethylation has been proposed to be carried out in two ways namely, active demethylation, with the enzymatic help of 5 mC hydroxylase (ten-eleven translocation 1, TET1) and also passive demethylation caused by the suppression of DNMTs during DNA replication [29,30]. Growth arrest and DNA damage (*Gadd45*) genes, involved in stress and growth arrest, have been shown to mediate repair-based DNA demethylation [31]; *Gadd45a* and *Gadd45b* have been proven to play crucial roles in DNA demethylation [32,33,34,35,36]. Methyl-CpG-binding proteins (MeCPs) are also important mediators of DNA methylation in the regulation of gene expression [26]. 

### 1.3. Histone Modifications

Histones are proteins which DNA is wrapped around. They package and order DNA into structural units known as nucleosomes, which are then condensed to form chromatin and subsequently further condensed into chromosomes [37]. Modifications to histone proteins include acetylation, methylation, phosphorylation, ubiquitination, sumoylation and adenosine diphosphate (ADP) ribosylation, with the most well-studied modification being acetylation, and result in either transcriptional activation or repression [14,38]. Histone acetylation involves the addition of an acetyl group from acetyl coenzyme A (acetyl-CoA), catalyzed by histone acetyltransferase (HATs). This results in the loose packing of nucleosomes, allowing transcription factors to bind to the DNA, bringing about transcriptional activation [39]. Histone acetylation is reversible, and the deacetylation of histone is catalyzed by histone deacetylases (HDACs). Histone methylation involves the addition of methyl groups to histone proteins, with lysine and arginine residues being the most common sites for methylation marks [40]. Lysine residues on histone proteins can be mono, di or tri-methylated [41]. Histone methylation can bring about either transcriptional repression or transcriptional activation. Trimethylation of histone H3 at lysine 4 (H3K4me3) is associated with transcriptional activation and is upregulated in the hippocampus during memory formation [42]. Histone phosphorylation involves the addition of phosphate groups to the histone tail, which may take place during cellular response to DNA damage when phosphorylated histone H2A(X) demarcates large chromatin domains around the site of DNA breakage [43]. The transport of ubiquitin to the histone core proteins H2A and H2B [44] (or modification of histone H4 by small ubiquitin-related modifier (SUMO) family proteins [45]) is defined as histone ubiquitination or histone sumoylation respectively. ADP-ribosylation (ADPr) is a reversible post-translational modification of proteins, which controls major cellular and biological processes, including DNA damage repair, cell proliferation and differentiation, metabolism, stress and immune responses [46].

### 1.4. Non-Coding RNA

Non-coding RNAs (ncRNAs) are functional RNA molecules transcribed from DNA but not translated into proteins [47]. Examples of ncRNAs include microRNAs (miRNAs), long non-coding RNAs (lncRNAs), short-interfering RNAs (siRNAs) and piwi RNAs (piRNAs). A predominant class of ncRNAs are miRNAs, which are small non-coding RNA molecules of about 22 nucleotides in length that bring about RNA silencing and post-transcriptional regulation of gene expression [48]. miRNAs bind to specific target messenger RNAs (mRNAs) with complementary sequences inhibiting their expression [49] by mRNA degradation or translational repression. Target mRNAs may be regulated by multiple miRNAs; similarly, miRNAs are able to regulate the expression of more than one target [50]. In addition to miRNAs, lncRNAs and piRNAs have also been shown to play roles in epigenetic regulation. Some lncRNAs are related to genomic imprinting and have been shown to be associated with chromatin-modifying complexes regulating gene transcription [51]. Similarly, piRNAs have been associated with chromatin regulation and are able to repress gene transcription; however, piRNAs have also been found to be involved in transposon methylation by the DNA methltransferase family (DNMT3a and DNMT3b) [52] and also DNA methylation in germ cells [53].

## 2. Epigenetic Regulation of Hippocampal Neurogenesis

The generation of new neurons in the adult central nervous system (CNS) in the subventricular zone (SVZ) of the lateral ventricle and the subgranular zone (SGZ) of the dentate gyrus (DG) in the hippocampus is one of the major breakthroughs in neuroscience research. Adult hippocampal neurogenesis occurs in the DG and refers to the formation of new functional dentate granule cells from neural stem cells (NSCs) [10] contributing to learning and memory [11] and also mood regulation [54]. Adult hippocampal neurogenesis was first observed in rodents [55] and later confirmed in humans [56,57,58]. This process occurs when intermediate neural progenitors (IPCs) are amplified and integrated into existing neural circuits. Due to this, adult hippocampal neurogenesis provides a means for both functional and structural plasticity in the hippocampus. Dysregulation of adult hippocampal neurogenesis has been shown to cause cognitive decline and psychological symptoms [10].

The process of adult hippocampal neurogenesis is regulated by factors that are both extrinsic and intrinsic [59], which are able to actively upregulate or downregulate the generation of new neurons throughout adulthood and may occur prenatally or postnatally. One of the key regulators of neurogenesis is epigenetics. Studies have shown that epigenetic regulators are crucial for the generation of neurons from adult neural progenitors that integrate into the hippocampus [8,26,60,61]. These neural progenitor cells have high levels of histone H3 lysine 4 trimethylation (H3K4me3) and histone H3 lysine 27 trimethylation (H3K27me3); alterations of H3K4me3 and H3K27me3 are often the focus of environmental epigenetic studies due to their strong association with gene expression at promoters. Histone deacetylase 1 (HDAC1) is mainly expressed in NSCs, while mature neurons mainly express histone deacetylase 2 (HDAC2) [62] suggesting that the expression of HDACs may be developmentally regulated. Combined deletion of both these HDACs resulted in an inability for neuronal precursors to differentiate into mature neurons, leading to excessive cell death [63]. In cases where HDAC2 was depleted, studies have shown that HDAC1 was able to compensate for the loss of HDAC2 [64], and thus, neurogenesis is not affected. In terms of histone acetylation, *KAT6B*, a gene which provides instructions for making histone acetyltransferases, is highly expressed in the adult SVZ, and a deficiency of *KAT6B* results in a decrease in NSCs [65].

Tritorax group (trxG) and polycomb group (PcG) proteins activate or silence gene expression respectively, through a chromatin remodeling system [66]. Epigenetic modifications of chromatin structure by PcG proteins, which function as transcriptional repressors, aid in the maintenance of cellular identity [67]. These proteins facilitate the trimethylation of the lysine 27 of histone 3 (H3K27me3), bringing about transcriptional repression, and trxG protein complexes catalyze the trimethylation of H3K4 (H3K4me3) [11]. Mixed-lineage leukemia I (Mll1) is a member of the trxG family and it is expressed in the SVZ and olfactory bulb (OB) [68]. Mll1 has been shown to be crucial for the proliferation and neurogenesis of SVZ and the olfactory bulb (OB) NSCs [66] and is known to be a histone methyltransferase (HMT) for histone H3 lysine 4 [69]. Studies have shown that a deficiency of Mll1 in the SVZ severely impaired neuronal differentiation [66].

Sex determining region Y-box 2 (SOX2) has been reported to prime the epigenetic landscape in neural progenitors, as the early SOX2-dependent imbalance in H3K4me3 and H3K27me3 marks has a profound impact throughout the entire differentiation process, which allows for proper gene activation during neurogenesis [8]. The epigenetic mechanisms that regulate neurogenesis are extremely strongly associated with each other and other regulatory pathways [70].

The involvement of DNA methylation is seen in studies with *Gadd45b*, which is a crucial component for the DNA methylation of certain promoters and their corresponding gene expressions essential for neurogenesis [60], thereby increasing the expression of key neuronal genes such as *Fgf1* and *Bdnf* [65]. Mice with *Gadd45b* deletions showed deficits in the proliferation of neural progenitors and dendritic growth in the hippocampus [60]. In addition, mice exposed to prenatal stress showed increased expressions of DNA methyltransferase 1 (DNMT1), and also an increase in its binding to the glutamic acid decarboxylase 67 (GAD67) promoter, leading to an impairment in the genesis of the gamma-aminobutyric acid (GABA) interneurons, suggesting that prenatal upregulation of DNMT1 may reduce interneuron genesis [10]. On the other hand, the expression of DMNT1 in neural precursor cells was also shown to be crucial for the survival of newly generated neurons in the adult hippocampus. Deletion of DNMT1 in NSCs at an early stage of DG development impaired the ability of NSCs to establish secondary radial glial scaffolds and to migrate into the SGZ of the DG, leading to aberrant neuronal production in the molecular layer, increased cell death and decreased granule neuron production. Furthermore, it promoted the differentiation of NSCs into astrocytes [71,72]. In addition to DMNT1, DMNT3-knockout mice were also found to have significantly fewer immature neurons [73]. DNMT1 has been shown to control the timing of astrogliogenesis through Janus kinase/signal transducers and activators of transcription (JAK-STAT) signaling, and DNMT3A and DNMT3B are required for neuron specification [65]. Maternal exposure to 3,3’-iminodipropionitrile (IDPN) was shown to affect hippocampal neurogenesis in offspring, and this was found to be due to hypermethylation of genes *Edc4*, *Kiss1* and *Mrpl38* [13]. DMNTs have been proven to be crucial in ensuring normal neurogenesis, as dysregulation or mutations in DNMTs result in abnormal neurogenesis [74] which further confirms the importance of DNA methylation in hippocampal neurogenesis. Reductions in hippocampal neurogenesis have also been linked to glucocorticoid hormones (GC) which regulate neural stem/precursor cell proliferation via changes in the methylation state of gene promoters associated with cell cycle regulation and Wnt signaling [75]. Disruption of GCs causes alterations in dendritic morphology and numbers and the appearance of new granule neurons. 

Methyl-CpG-binding protein 2 (MeCP2) is a member of the methyl-CpG binding domain (MBD) family that plays an important role in both neuronal and astrocytic lineage specification by repressing astrocytic genes during neurogenesis and releasing this repression during astrogenesis [76]. MeCP2 appears to play a key role in conveying neuronal signaling and activity into epigenetic gene regulation. MeCP2-knockout mice have neurons with smaller nuclei and fewer dendritic branches [77], and activity-dependent changes in DNA methylation were associated with a reduction in MeCP2 binding [78]. MeCp2 deficiency also correlated with poor neural progenitor cell (NPC) maturation and impaired dendritic and spine morphogenesis in new neurons [79]. In addition to MeCP2, MBD1 proteins of the MBD family have also been shown to play crucial roles in hippocampal neurogenesis [70], and it is their MBD domains that interact with methylated CpG, and thus, MBD binding correlates with DNA methylation. MBD1-deficient mice had lower levels of neurogenesis and impaired spatial learning capabilities [80] and were also found to be susceptible to depression [81]. It is evident that DNA methylation has a crucial role in the maintenance of NPCs and their fate specification in adult hippocampal neurogenesis.

It has also been shown that MeCP2 is able to epigenetically regulate miRNAs in adult NSCs, which brings in another form of epigenetic regulation. Co-regulation of miR-137 together with SOX2 regulates the proliferation and differentiation of adult neural stem cells [82]. Knockdown of miR-137 enhances their differentiation while overexpression of miR-137 promotes their proliferation. Furthermore, miR-137 has been found to repress the expression of enhancer of zeste homolog 2 (EZH2), a PcG histone methytransferase, which brings about a global reduction in H3K27me3-modulated neurogenesis [82]. Similarly, high levels of miR-184 were found to promote proliferation of neural progenitors while inhibiting their differentiation [83].

**Figure 1:**
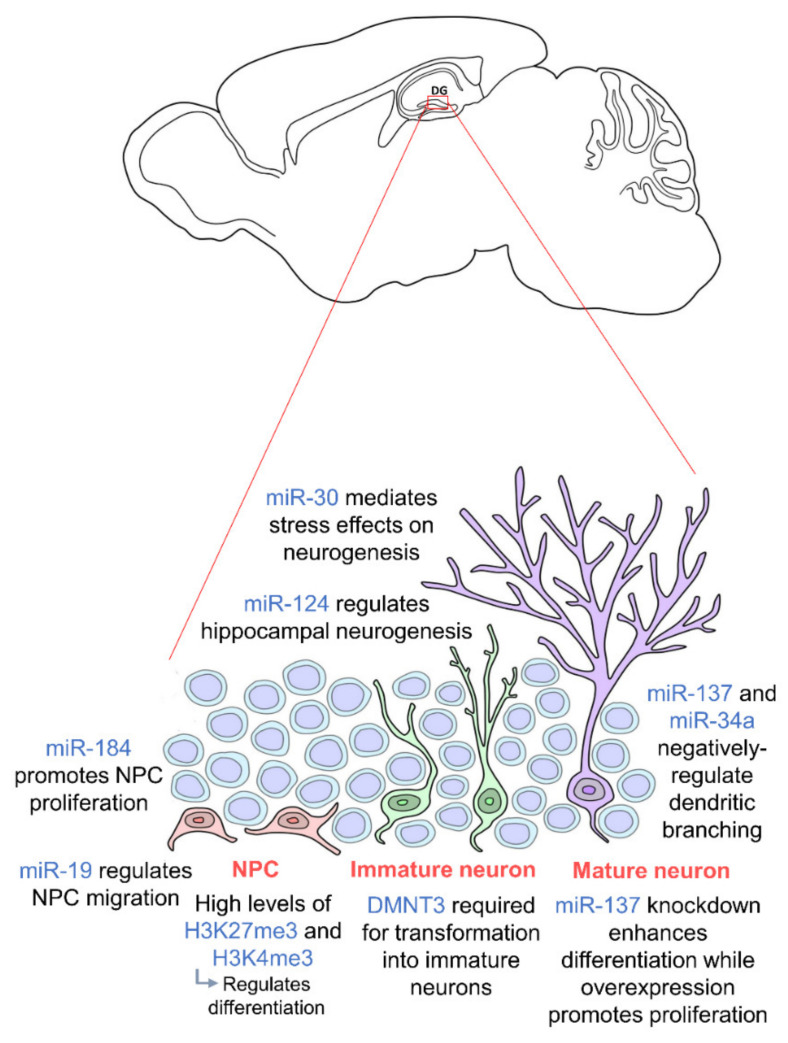
Epigenetic regulation during adult hippocampal neurogenesis. Adult hippocampal neurogenesis is regulated by epigenetics at every stage. Neural progenitor cells (NPCs) (orange) require high levels of H3K27me3 and H3K4me3 for proper differentiation, and their proliferation and migration are regulated by miR-184 and miR-19 respectively. In order for NPCs to transform into immature neurons (green), DMNT3 is required. Mature neurons (purple) are then regulated by miR-137 and miR-34a. miR-30 and miR-124 regulate adult hippocampal neurogenesis in general. NPCs are located in the subgranular zone (SGZ) of the dentate gyrus, and mature neurons integrate into the granule cell layer. (DNMT: DNA methyltransferase).

The miR-30 family of miRNAs has also been shown to mediate the effects of stress on hippocampal neurogenesis in mice [84]. These miRNAs were found to be downregulated in stressed mice and involved in neural progenitor cells differentiation. In addition, miR-137 and miR-34a have been shown to negatively regulate dendritic branching and the complexity of newborn neurons [85,86], and miR-19 has been proven to be crucial for the migration of these newborn neurons [87]. Human neural progenitor cells with abnormal expression of miR-19 display deviant migration patterns. Regulation of ten-eleven translocation protein 1 (TET1), a methylcytosine dioxygenase, and miR-124 has also been shown to modulate hippocampal neurogenesis. Together with miR-9, miR-124 appears to repress Brg- and Brahma (Brm)- associated factor-complex 53a (BAF53a) allowing neural progenitors to properly differentiate into neurons [69]. Thus, miR-124 is very lowly expressed in progenitor cells but upregulated in differentiation and mature neurons [88]. TET1 controls the demethylation of miR-124 thereby regulating its expression. Dysregulation of TET1 and miR-124 due to Down Syndrome critical region 1 (DSCR1) protein knockout led to an impairment in hippocampal neurogenesis [89]. TET1 is also known to interact with MeCP2 further confirming the importance of epigenetic regulation including the crosstalk between different epigenetic factors [90]. Several studies also show that a synergistic modulation by various different miRNAs facilitates hippocampal neurogenesis and is crucial for neurogenic lineage fate determination in the adult hippocampus [91,92,93]. Epigenetic factors governing adult hippocampal neurogenesis are summarized in Figure 1. We are just beginning to understand the influence of epigenetics on hippocampal neurogenesis as an intermediate regulatory mechanism between DNA sequences and gene expression. Thus, further studies should be carried out on the molecular pathways to induce, remove and interpret epigenetic modifications. Looking downstream, this may enable us to further elucidate the mechanisms of neurodevelopment-related disorders and aid the development of novel therapeutic approaches to prevent abnormal brain development.

## 3. Epigenetic Regulation of Hippocampal Principal Neurons

The two principal neuronal cell types in the hippocampus are pyramidal cells and granule cells [3]. These pyramidal and granule cells form an intrinsic circuitry making up the hippocampal formation. Neurons in the hippocampus undergo long-term potentiation (LTP), a process believed to be the basis of neuroplasticity that underlies memory formation [94]. Nevertheless, the ability of the brain to code information pertaining sensory, spatial or temporal changes in our everyday lives is made possible through hippocampal neurons. Studies have even gone so far as to suggest that hippocampal neurons may organize our experiences into distinct, transferable units [95]. Animal studies have shown that hippocampal principal cells are developmentally regulated by a characteristic DNA methylation program (DMP), including 5-methylcytidine (5 mC), 5-hydroxylmethylcytidine (5 hmC) and their binding proteins, leading to the hippocampal neuronal differentiation and maturation spatiotemporally, as indicated by their phenotypic marks in the cornus ammonis (CA) and the DG prenatally and postnatally [96]. As hippocampal neurons form the basis of learning and memory, epigenetic factors that modulate neuronal function will inevitably modulate memory formation as well. In neurons, acetylation of histones is dependent on acetyl-CoA produced from acetate and this regulates gene expression. Acetyl-CoA synthetase 2 (ACSS2), the enzyme that facilitates this process, has been shown to be a direct regulator of histone acetylation in neurons [9]. ACSS2 is localized in the nucleus of hippocampal neurons, and inhibition of ACSS2 leads to a decrease in histone acetylation and the impairment of long-term spatial memory, a process reliant on histone acetylation [9]. Extracellular stimulation of hippocampal neurons in rat brains show that histone acetyltransferase (HAT) Tip60 translocates to the nucleus, where it mediates the epigenetic control of genes that allow for chromatin reorganization in the hippocampus [97]. In studies where there was an inhibition of DNMTs in the hippocampus of rats, there was an inability to consolidate memories following fear conditioning [98]. DNMT inhibition was also found to prevent the induction of LTP suggesting that DNA methylation may govern memory formation.

**Figure 2:**
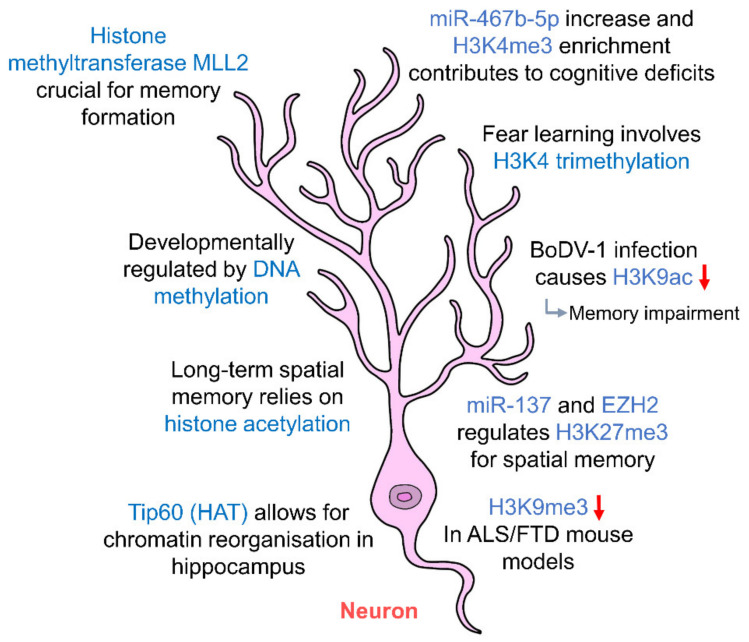
Epigenetic regulation of hippocampal neurons. Hippocampal neurons are regulated by histone acetylation and methylation which in turn affect long-term memory. DNA methylation regulates the development of hippocampal neurons. Abnormal changes to the levels of histone marks contribute to cognitive deficits and memory impairment. (HAT: histone acetyltransferase; ALS: amyotrophic lateral sclerosis; FTD: frontotemporal dementia).

Methylation of histones proves to be equally important in the epigenetic regulation of hippocampal neurons (Figure 2, Table 1). Acute stress has been shown to increase the expression of repressive histone mark, H3K9me3 in CA1, CA3 and DG neurons of the hippocampus [99], resulting in changes in gene expression and circuit connectivity. Histone methyltransferase MLL2 (KMT2B) has also been shown to be crucial for memory formation in mice [100]. In mice lacking KMT2B, DNA microarray analysis showed a downregulation of 152 genes in the DG of the hippocampus and significant decreases in H3K4me3 and H3K4me2 levels resulting in impaired memory function. Methylation of histones has also been shown to facilitate long-term memory formation in the hippocampus, as fear learning induced an upregulation of H3K4 trimethylation in hippocampal neurons as early as 1 h post-conditioning [101].

Infections or diseases that alter histone acetylation/methylation levels have also been shown to impair memory. An example of this is in the case of Borna disease virus 1 (BoDV-1) which brings about a reduction in H3K9 acetylation upon infection, leading to memory impairment [102]. In amyotrophic lateral sclerosis (ALS) and frontotemporal dementia (FTD) mice models, there was a marked decrease in H3K9me3 staining in hippocampal neurons, leading to neuronal loss in CA1, CA3 and DG regions and cognitive dysfunction related to the hippocampus [103]. In addition, primary hippocampal neurons exposed to ketone metabolite β-hydroxybutyrate (BHBA) brought about an upregulation of H3K27 acetylation at Bdnf promoters and also a decrease in H3K27 trimethylation, ultimately activating *Bdnf* transcription [104]. Brain-derived neurotrophic factor (BDNF) is one of the key regulators of synaptic plasticity and memory formation [105], and the ability of histones to regulate *Bdnf* transcription highlights their influence on memory and synaptic plasticity.

In addition to histone modifications, miRNA targeting has also been proven to be an epigenetic regulator of hippocampal neurons. Studies have shown the miR-137 and EZH2 regulate H3K27me3 levels and a disruption in this regulation leads to spatial memory deficits [106]. EZH2, a methyltransferase, also regulates the mammalian target of rapamycin (mTOR) signaling pathway by altering H3K27me3 levels and DNA methylation in the phosphatase and tensin homolog (PTEN) coding and promoter regions [107] necessary for memory consolidation. Both mTOR and histone methylation are crucial for hippocampal consolidation of memories [100,101,108]. Vesicular glutamate transporter 2 (VGLUT2) is a glutamate transporter that allows uptake of glutamate into synaptic vesicles. Studies have shown that prenatal exposure to ethanol upregulates VGLUT2 expression in adult hippocampal neurons associated with decreased DNA methylation changes in the promoter, enrichment of H3K4me3 and also an increase in miR-467b-5p levels [109]. These epigenetic modifications altering glutamate neurotransmission in the hippocampus are said to contribute to deficits in cognition and behavior. These studies further emphasize the importance of epigenetic regulation in all forms, be it histone modifications, DNA methylation or miRNAs targeting in the modulation of hippocampal neurons and subsequently synaptic transmission in learning and memory. Our current understanding of the epigenetic regulation of hippocampal pyramidal cells and granule cells is still in its infancy. It may be interesting to further explore how epigenetic regulation affects neurotransmitter release and uptake, and neuromodulators and their receptor systems. The influences these epigenetic mechanisms have on neurotransporters and ion channels in principal neurons in the hippocampus, and how they may affect cognition and memory, are also worth investigating.

## 4. Epigenetic Regulation of Hippocampal Interneurons

Hippocampal interneurons are GABAergic neurons that control the inhibitory activity within the hippocampus. These interneurons account for approximately 10–15% of the total neuronal population [110] and carry out their functions through the release of the neurotransmitter GABA. There are various different subtypes of interneurons, namely, axo-axonic cells (AACs) also known as Chandelier cells, parvalbumin-expressing basket cells (PVBCs), bistratified cells (BiCs), cholecystokinin-expressing basket cells (CCKBCs), oriens–lacunosum-moleculare cells (O-LMs), neurogliaform cells (NGFCs) and ivy cells (IvCs) [110]. These GABAergic neurons innervate excitatory neurons and enable the balance of excitation and inhibition to be achieved [111]. This dynamic interplay between principal neurons and interneurons underlies information processing in the hippocampus.

Hippocampal interneurons have also been shown to be epigenetically regulated (Table 1). These interneurons have been observed to have distinct DNA methylation patterns regardless of phenotypic similarity, indicating diverse epigenotypes within similar neuronal phenotypes in different microcircuits in the hippocampus [112]. Long non-coding RNA (lncRNAs) are another type of non-coding RNA, and it was found that in lnc-RNA-Evf2-knockout mice, the number of interneurons was significantly reduced [113]. Evf2 recruits distal-less homeobox (DLX) and MeCP2 transcription factors, which are crucial for DNA regulation and expression, ultimately regulating the formation of hippocampal GABAergic interneurons [14]. In addition, the lack of lnc-RNA-Evf2 appeared to induce epilepsy [14], which indicates a lack of GABAergic interneurons or dysfunction allowing for runaway excitation [114]. This suggests that adequate production of GABAergic interneurons in the hippocampus is reliant on non-coding RNA-dependent gene regulation and is therefore epigenetically regulated.

GABAergic interneurons are key modulators of adult hippocampal neurogenesis. In prenatally-stressed mice, epigenetic modifications of GABAergic interneurons cause depression and also deficits in hippocampal neurogenesis [10] due to an increase in DNMT1 binding to the GAD67 promoter. Mice exposed to prenatal stress had higher levels of DNMT1 and DMNT3a as compared to controls, and this overexpression of DNMTs correlated with a decrease in reelin and GAD67 expression [115]. These mice also showed increased binding of DNMT1 and MeCP2, and increased DNA methylation in CpG-rich regions of reelin and GAD67 promoter regions, resulting in hyperactivity and impaired social interaction abilities. DNMT1 levels in GABAergic interneurons have been shown to be affected by nicotine from tobacco smoking. Nicotine was found to decrease DNMT1 mRNA and protein levels in mice given four nicotine injections [116]. It was also found that maternal exposure to hexachlorophene (HCP) brought about a disruption in neurogenesis in the hippocampus, and this was due to a reduction in the number of GABAergic interneurons as a result of hypermethylation of distal-less homeobox 4 (DLX4), doublesex and mab-3-related transcription factor 1 (DMRT1) and phospholipase C beta 4 (PLCB4) [12]. These studies show that the GABAergic interneurons of the hippocampus are indeed epigenetically regulated and that they are key modulators in hippocampal neurogenesis. So far, there has been no report of modifications to histone proteins in the hippocampal interneurons. Furthermore, as the hippocampal interneurons exhibit many different morphological phenotypes with varying modes of discharge, understanding the epigenetic modulations of each type of interneuron, in particular, histone modifications, may shed some light on the excitatory and inhibitory changes in hippocampal neurons and relevant neurological and neuropsychological disorders. 

## 5. Epigenetic Regulation of Hippocampal Glial Cells (Astrocytes, Microglia and Oligodendrocytes)

Glial cells are the non-neuronal cells which make up approximately 80% of the cells in the human brain [117], suggesting their influence and importance to neurons, the main players of memory formation and cognition. Glial cells in the CNS consist of microglia, oligodendrocytes and astrocytes [118], and this is also true of the hippocampus [119]. They play key roles in the housekeeping activities of the brain, providing protection and support and maintaining homeostasis in the CNS. While once thought to only play minor supporting roles in the housekeeping of the brain, they have now been shown to be crucial in regulating synaptic plasticity, transmission and memory formation [119,120,121]. In the hippocampus, glial cells have long been shown to play integral roles in the regulation of neuronal connections and synaptic transmission [119,122,123,124], and this finding has not changed over time but has become more and more evident. Thus, it is not surprising to find that glial cells of the hippocampus are also regulated by epigenetic factors (Table 1).

### 5.1. Astrocytes

Astrocytes are the most abundant of all glial cells and promote neuronal survival [125] and maintenance through the uptake and release of extracellular ions [126]. They are mainly found in the hippocampus and cerebral cortex with ramified cell bodies allowing contact with hippocampal neurons and their synapses [127]. In addition to neurons being epigenetically regulated, the study by Jury et al. (2020) also showed a significant decrease in H3K9me3 staining in astrocytes in C9ALS/FTD BAC mouse models (C9BAC) [103], which may contribute to cognitive deficits seen in ALS. Astrocytes have been shown to protect neurons in the hippocampus by potassium buffering through an inward-rectifying potassium channel Kir4.1 [128]. The expression of Kir4.1 in astrocytes has been demonstrated to be regulated by DNA methylation through DNMT1 [129], indicating that DNA epigenetic regulation of astrocytes confers neuronal protection in the hippocampus. In addition, in Alzheimer’s disease (AD) patients, H2A histone family member X (H2AX) was seen to be phosphorylated more as compared to control [130], further alluding that hippocampal astrocytes are epigenetically regulated.

**Figure 3:**
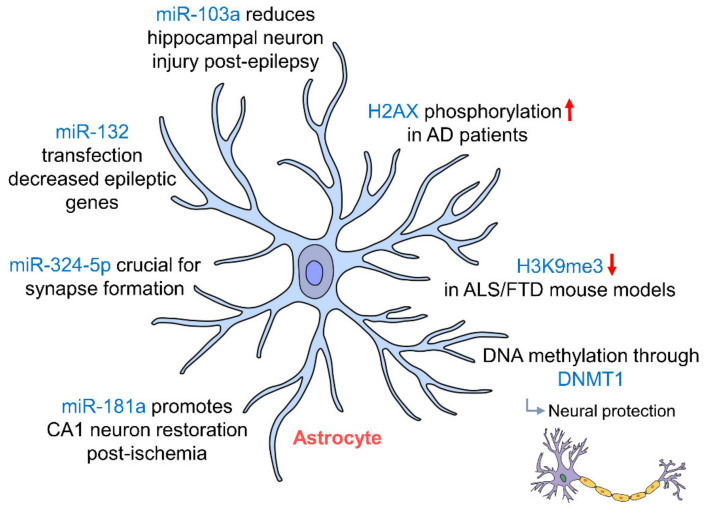
Epigenetic regulation of hippocampal astrocytes. Astrocytes in the hippocampus protect neurons via potassium buffering through Kir4.1 channels, which are regulated by DNA methylation through DNMT1 miR-103a, miR-181a and miR-132 regulation; astrocytes protect neurons post-injury by preventing astrocytic activation and promote the restoration of CA1 neurons in the hippocampus. (AD: Alzheimer’s disease; ALS: amyotrophic lateral sclerosis; DNMT1: DNA methyltransferase 1; FTD: frontotemporal dementia).

Astrocytic miR-324-5p has been shown to be crucial in facilitating the formation of synapses due to the suppression of chemokine ligand 5 (CCL5). Presynaptic marker synapsin 1 (SYN1) was found to be reduced by 79.1% in Dicer-knockout mice [131]. In epileptic mouse models, inhibition of miR-103a prevented the activation of astrocytes in the hippocampus allowing for a reduction in hippocampal neuron injury following epilepsy [132]. Death of astrocytes from these epileptic seizures also seemed to be epigenetically regulated by miR-34b-5p, which directly targets B-cell lymphoma 2 (Bcl-2) modulating apoptosis [133]. There was also an increase in miR-132 expression in glial cells in both rat and human epileptogenic hippocampus. Transfection of miR-132 in human primary astrocytes brought about decreases in the expression of genes involved in epileptogenesis, such as *COX-2*, *IL-1β*, *TGF-β2*, *CCL2* and *MMP3* [134]. Following ischemic injury, inhibition of miR-181a in astrocytes has been shown to promote the restoration of CA1 neurons in the hippocampus in rats [135]. These studies show the extent to which astrocytes in the hippocampus are epigenetically regulated (Figure 3, Table 1). While different epigenetic regulations of astrocytes have been documented through physiological and pathophysiological changes of hippocampus, how these epigenetic regulations affect astrocytic glutamate transporter, ion channels and gap junction proteins remains unknown. Further study in these areas may provide us with a better understanding of the effects of transcriptional activation and repression on hippocampal functional changes and disease genesis.

### 5.2. Microglia

Microglia are the macrophages of the CNS and serve as the first line of defense against injury or insult, and make up roughly 5–12% of the total CNS cell population [136]. In a healthy adult brain, the ramified microglia act as surveillance cells [137], as their processes are highly motile and they are able to extend and retract to actively scan and survey the brain’s parenchyma [138]. In the hippocampus, microglia are key players in adult hippocampal neurogenesis [139,140,141]. They shape hippocampal neurogenesis by means of apoptosis-coupled phagocytosis [142]. Hippocampal microglia have been said to be more vigilant than in other parts of the brain [143] and in the cerebellum.

Microglia have been shown to be epigenetically regulated by histone modifications and also post-translationally regulated by sumoylation [120]. Inhibition HDACs brought about an enrichment H3K9ac in the promoter region of microglial phosphatidylinositol 3-kinase (PI3K) [120], a key player in neuronal long-term potentiation (LTP) [144]. The effects of HDAC inhibition on the PI3K/Protein kinase B (AKT) pathway ultimately affect the expression of microglial BDNF, which has been shown to act on neurons and alter synapse formation and transmission [145]. Changes in microglial BDNF expression were found to alter neuronal LTP [120,146] signifying the possibility of using epigenetics to regulate LTP and memory in the hippocampus via microglia. Suberoyl anilide hydroxamic acid (SAHA), another histone deacetylase inhibitor, was found to reduce toll-like-receptor 4 (TLR4)/myeloid differentiation primary response protein 88 (MYD88) signaling in hippocampal microglia through an increase in histone acetylation [147], which may be protective in the case of epileptic brain damage. HDACs 1 and 2 have also been shown to modulate microglial function in the hippocampus and cortex in the processes of homeostasis, development and also neurodegeneration [148]. 

**Figure 4:**
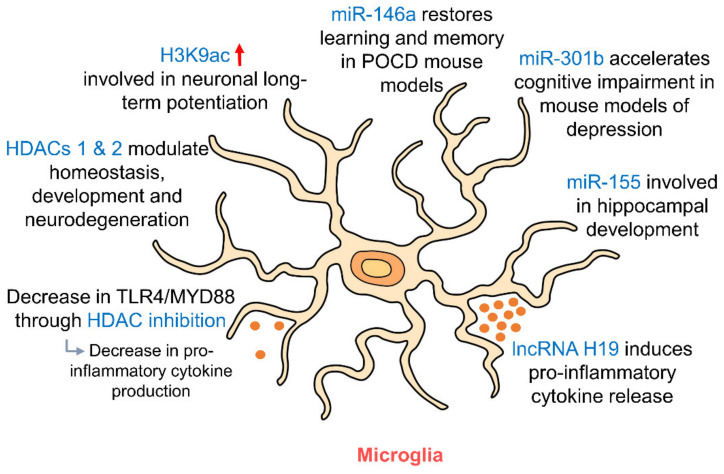
Epigenetic regulation of hippocampal microglia. As resident immune cells of the brain, microglia release pro- and anti-inflammatory cytokines depending on their polarization. HDAC inhibition and RNA targeting influence this release of cytokines. HDACs 1 and 2 in microglia regulate their involvement in homeostasis, development and neurodegeneration. miR-146a, miR-301b and miR-155 in microglia are involved in regulating memory and cognition and in hippocampal development. (HDAC: histone deacetylase; MYD88: myeloid differentiation primary response 88; POCD: postoperative cognitive dysfunction; TLR4: toll-like receptor 4).

In addition to histone modifications, microglia have also been shown to be epigenetically regulated by miRNAs (Figure 4, Table 1). Overexpression of miR-301b was found to accelerate hippocampal microglial activation and cognitive impairment in mice models of depression [149] suggesting a role for epigenetic regulation of hippocampal microglia in the pathophysiology of mood disorders. miR-155 was also shown to be involved in inflammation-induced neurogenic deficits through microglial activation which caused abnormal development of the hippocampus [150]. In the postoperative cognitive dysfunction (POCD) mouse model, miR-146a was able to restore learning and memory impairment by suppressing hippocampal neuroinflammation via microglia [151]. Looking upstream—the removal of Dicer, the ribonuclease that is crucial for miRNA biogenesis—from microglia both prenatally and postnatally was found to have profound effects on the hippocampus, whereby Dicer-negative microglia showed compromised hippocampal neuronal function [152]. The effects of most miRNAs on microglia appear to be related to the NF-κB pathway [153], suggesting a central role for this pathway in the epigenetic regulation of microglia by miRNAs. Other than miRNAs, lncRNA H19 has also been shown to contribute to both hippocampal microglial and astrocytic activation, leading to a release of pro-inflammatory cytokines [154] via the JAK/STAT signaling pathway. These studies suggest an influential role of non-coding RNAs in the epigenetic regulation of microglia and astrocytes in the hippocampus. Studies have suggested that AD may be at least partly attributed to global DNA methylation changes in microglia [155]. In addition, *IL1β* gene expression is regulated by DNA methylation in aging microglia [156]. Recent studies have brought to light the possibility of microglia playing a far bigger role in memory processing and synaptic plasticity than we once thought [120]. Further studies on the regulatory influences of epigenetics on microglia by DNA methylation, histone modifications or miRNA targeting may shed more light on the involvement of microglia in memory formation and may allow us to modulate synaptic plasticity via epigenetic regulation of microglia.

### 5.3. Oligodendrocytes

Oligodendrocytes are the myelinating cells of the CNS which insulate axons facilitating the conductance velocity of action potentials [157]. Oligodendrocyte precursor cells (OPCs) originate from the ventricular zones of the brain and spinal cord, and from there they migrate throughout the developing CNS where they differentiate into myelinating oligodendrocytes [158]. They are found more abundantly in the CA2 region of the hippocampus as compared to CA1 and CA3 areas [159], suggesting a role in social memory [160]. Oligodendrocytes respond to neuronal activity with prolonged membrane potential depolarization, which facilitates the conduction velocity of action potentials along the myelin sheaths of axons in the hippocampus [161].

**Figure 5:**
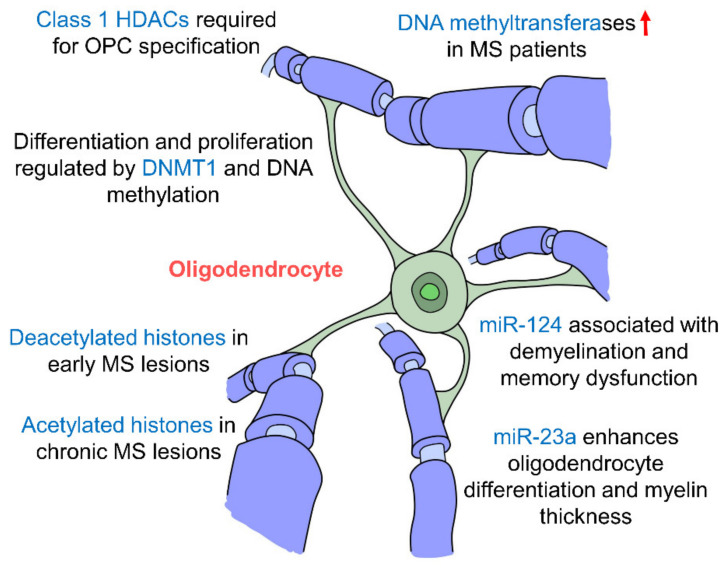
Epigenetic regulation of hippocampal oligodendrocytes. Oligodendrocytes are the myelinating cells of the CNS. Changes in epigenetic factors such as DNA methylation, histone modifications and miRNA regulation affect myelination of neurons and are seen in the multiple sclerosis (MS) pathology. Oligodendrocyte differentiation and specification are also affected by these epigenetic changes by miR-23a, miR-124, DNMT1 and Class 1 HDACs. (DNMT: DNA methyltransferase; HDAC: histone deacetylase; MS: multiple sclerosis).

Studies have found that early postnatal exposure of isoflurane brings about a decrease in the expression of DNMT1 and DNA methylation in oligodendrocytes in the hippocampus, inhibiting their differentiation and proliferation [162]. In fact, hippocampal demyelination along with memory impairments are several hallmarks of multiple sclerosis (MS), an autoimmune demyelinating disease of the CNS. Studies have shown that MS patients had upregulated levels of DNA methyltransferases (DNMT1, DNMT3A and DNMT3B), and demethylation enzymes were decreased [163]. The loss of myelin correlated with changes in DNA methylation and four oligodendrocyte-specific genes were hypermethylated suggesting that oligodendrocytes are epigenetically regulated by DNA methylation [163]. A large number of oligodendrocytes with deacetylated histones were also seen in early MS lesions but interestingly in patients with chronic MS, there appears to be a shift towards high levels of histone acetylation [164] suggesting that histone deacetylation occurs in the early stages of MS but decreases as the disease progresses. Histone modifications have also been proven to be crucial in oligodendrocyte development whereby the inhibition of class I HDACs by valporic acid (VPA) was found to inhibit the specification of OPCs from NPCs [165]. More specifically, HDACs 1, 3 and 10 have been shown to promote oligodendrocyte differentiation [166]. miR-23a overexpression has been seen to enhance oligodendrocyte differentiation and myelin thickness via the mTOR/AKT cascade [167]. In addition, miR-124 has been shown to be associated with demyelination in the hippocampus and memory dysfunction [168], which suggests that miRNAs affect myelin integrity particularly in the hippocampus which may then cause impairments in memory formation further indicating the hippocampal oligodendrocytes are epigenetically regulated (Figure 5, Table 1). The epigenetic regulatory mechanisms that govern oligodendrocytes and their interactions with other cell types have mostly been studies in the context of MS. It would be interesting to explore how epigenetic changes in oligodendrocytes may affect memory formation in the hippocampus. In addition to oligodendrocytes, their precursors, OPCs, encompass approximately 3–4% of cells in the grey matter and 8–9% in the white matter, making them the fourth largest group of glia after astrocytes, microglia and oligodendrocytes [169] and the fifth largest cell population in the brain. OPCs are particularly prevalent in the hippocampus [170], where they interact with neurons, astrocytes and microglia, and may also be able to differentiate into neurons and astrocytes [171]. Thus, it may be worthwhile to further study the epigenetic regulation of OPCs and the downstream effects this may have in the hippocampus.

## 6. Radiation-Induced Epigenetic Changes in the Hippocampus

Environmental toxicants can alter epigenetic regulatory features of the hippocampus leading to cognitive impairment. With increasing building of nuclear power plants to produce clean energy to reduce environmental contamination, and the use of ionizing radiation in modern medical diagnosis and treatment and space travel, the negative impacts of radiation exposure to human health have now attracted significant attention. Radiation induces DNA damage, and therefore, it is not surprising that it would also induce a cascade of epigenetic changes such as DNA methylation and affect the expression of DNA methyltransferases [172]. Extensive studies on the relationship between radiation damage and epigenetic mechanisms in recent years suggest that histone modifications, DNA methylation and miRNA targeting may be associated with the effects of irradiation [15]. While ionizing radiation (IR) is commonly used on many imperative diagnostic and treatment methods, it is also known to cause many unwanted side effects such as DNA damage [173]. IR can also trigger changes in the expression of genes and the control of cell cycles, and disruptions in mitochondrial processes and apoptosis. Cranial radiation therapy has been found to cause side effects in the CNS, such as declines in memory function, cognitive abilities and attention [173]. In young patients, it has been shown to hinder normal brain development and possibly lead to neuropsychological or psychosocial outcomes [5]. 

Proton irradiation was found to cause significant long-term epigenetic effects on the hippocampus of mice whereby there was an increase in 5 hmC 22 weeks following a single exposure to proton irradiation [174]. This shows the profound and long-term effects of a single proton irradiation on DNA methylation patterns specifically in the hippocampus. In another study by Torres et al. (2019), proton irradiation (1 Gy of 150 MeV) of mice upregulated individual and total amino acid synthesis in the hippocampus, whereas amino acid tRNA synthetase methylation was mostly downregulated [175]. The epigenetic changes in cytosine methylation (5 mC) and hydroxymethylation (5 hmC) in the hippocampus following ^56^Fe ion and proton were also observed which correlated with animal behavioral changes [176]. Furthermore, a novel class of DNA methylation change was observed following an environmental challenge, characterized by both increased and decreased 5 hmC levels along the entire gene body [177]. Other hippocampal-specific changes that occur due to irradiation include an inhibition of postnatal neurogenesis in the SGZ of the DG [178]. Space relevant irradiation was also seen to bring about cognitive impairment, resulting in a diminished ability to perform hippocampal-dependent behavior tasks, which correlated with significant increases in 5 mC and 5 hmC levels in the hippocampus, and increases in the levels of DNA methylating enzymes, such as TET1, TET3 and DNMT3a [179]. It has been suggested that overexpression of TET1 in the DG of the adult hippocampus may lead to an increase in the global levels of 5 hmC [180,181], and TET1 overexpression in CA1 area has been demonstrated to impair contextual fear conditioning [182,183]. These studies indicate that 5 mC, 5 hmC and TET may serve not only as radiation-induced epigenetic biomarkers, but also as parameters for evaluating the radioprotective effectiveness of drugs.

Whole brain irradiation with 2 and 30 Gy brought about a significant decrease in histone H3 acetylation and an increase of HDAC1 in hippocampus of rats at 7 and 30 days after radiation exposure [184]. This suggests that epigenetics is involved in irradiation-induced memory deficiency and alterations in chromatin structure may be a new possible molecular correlation of irradiation-induced cognitive deficiency [184]. Interestingly, HDAC2 expression was not affected by the irradiation. Irradiated rats also had profound memory impairments in the Morris water maze test and passive-avoidance test, which suggests that radiation-induced histone modifications in the hippocampus may ultimately lead to deficits in memory formation and cognitive function. Kang et al. (2017) assessed changes of enzymes associated with the epigenetic modifications of gene expression, including DNMT1, HDAC1, HDAC2, Sirtuin 1 (SIRT1) and acetylated histone H3 (Ace-H3) in the mouse hippocampus 1 and 30 days after acute cranial irradiation with 10 Gy [185]. They saw a downregulation of mRNA levels of HDAC1 1 day post-irradiation and decreases in mRNA levels of DNMT1, HDAC1 and HDAC2 at 30 days post- irradiation [185]. DNMT1, HDAC1, HDAC2, SIRT1 and Ace-H3 protein were reduced 1 and 30 days after irradiation with 10 Gy. This suggests that the reduction in epigenetic gene expression is associated with hippocampal dysfunction in mice exposed to cranial irradiation [185] with varying effects dependent on the time post-irradiation.

Functionally, the DNA methylation data appear to be consistent with involvement of post-synaptic mechanisms as effects of proton irradiation where the enrichment for 5 hmC changes near NMDA-subtype glutamate receptors links to LTP [177], leading to changes of postsynaptic neuronal function, as it has been shown that DNMT inhibition in hippocampal neurons results in activity-dependent demethylation of genomic DNA and a parallel decrease in the frequency of miniature excitatory postsynaptic currents (mEPSCs), which in turn impacts neuronal excitability and network activity [186]. Changes in NMDA receptor composition and postsynaptic density protein 95 levels, and an increase in the rate of miniature excitatory postsynaptic currents in the CA1 hippocampal region in response to radiation in rodent models and in human patients further support the involvement of radiation-induced DNA methylation and hippocampal neuronal function changes, and subsequent learning and memory impairment [187,188,189,190]. Increased GABA release from the cannabinoid type 1 receptor (CB1)-expressing basket cells (CB1 BCs) onto pyramidal cells after proton irradiation may be useful as a potential therapeutic target to prevent radiation-induced cognitive impairment [191]. In addition, epigenetic modulation via increased levels of microRNAs such as miR-34c, miR-488 [192], miR-132/miR-212 and miR-134 [193] in the hippocampus has also been reported after low and moderate cranial doses of radiation [193]. Furthermore, Kempf et al. (2014) found that the irradiation-induced alteration of miR-132 may result in rapid dendritic spine and synapse morphology alterations via aberrant cytoskeletal signaling and processing, leading to neurocognitive side effects observed in patients treated with ionizing radiation [193]. Interestingly, the total abdominal irradiation-induced elevated peripheral blood miR-34a-5p targeted the 3′UTR of brain-derived neurotrophic factor (*Bdnf*) mRNA in hippocampus to mediate cognitive dysfunction [194].

Non-ionizing radiation such as microwave exposure (30 mW/cm^2^) also induced an upregulation of 12 miRNAs and a downregulation of 14 miRNAs in the hippocampus of rats. These miRNAs are involved in brain-related signaling pathways, such as synaptic vesicle cycle, long-term depression, calcium signaling and neurotrophin signaling pathways [195]. A recent study has also shown that radio frequency radiation from mobile phone signals brought about a decrease in global DNA methylation and an increase in histone methylation in the rat hippocampus. These changes are dose- and frequency-dependent [196]. Therapeutic approaches by inhibiting methylation using 5-iodotubercidin [179], increasing 5 hmC and TET2 through force running to alleviate hippocampal cognitive deficits induced by radiation further confirm the important roles epigenetics may play in radiation-induced cognitive impairment [197]. Triggering of BDNF-TrkB signaling by inhibition of HDAC-1 may also stimulate neurogenesis as it has been reported that the high dose irradiation-induced decrease in histone deacetylase 1 (HDAC1)-dependent H3 acetylation is associated with long-term impairment of neurogenesis in the DG [198].

## 7. Concluding Remarks

Dissection of the epigenetic regulation of each type of hippocampal neuron and glial cell indicates significant ncRNAs—in particular miRNAs—in the regulation of hippocampal cells in addition to DNA methylation and histone modifications. Radiation exposure induces the abnormally epigenetic regulation of hippocampal neurons, which may result in rapid dendritic spine and synapse morphology alterations via aberrant cytoskeletal signaling and processing leading to neurocognitive side effects. As we are just beginning to understand the influence of epigenetics on hippocampal cells and function, further in-depth studies in the following areas may still be needed. (1) Molecular pathways to induce, remove and interpret epigenetic modifications of neurogenesis in the subgranular zone of the DG. (2) Epigenetic regulation of neurotransmitter release and uptake, and neuromodulators and their receptor systems in hippocampal neurons. (3) Epigenetic modulation of each type of hippocampal interneuron—particularly regarding neurotransmitter synthesis and release. (4) Epigenetic regulation of astrocytic glutamate transporter, ion channels and gap junction proteins. (5) Regulatory influences of epigenetics on the microglial involvement in memory formation and (6) epigenetic regulatory mechanisms that govern OPCs. As irradiation induces abnormally epigenetic regulation of neurons, it is reasonable to investigate whether similar changes occur in hippocampal glial cells, i.e., astrocyte, microglia, oligodendrocytes and oligodendrocyte precursor cells. Furthermore, by targeting those radiation-induced abnormal changes in miRNA, DNA methylation and histone modifications, novel therapeutic approaches may be developed to prevent radiation-induced acute and chronic hippocampal damages. Given that non-ionizing radiation could induce decreased global DNA methylation, an increase in histone methylation and upregulation of some miRNAs in the animal hippocampus, further study of low dose (<100 mSv) ionizing radiation-induced epigenetic changes in the hippocampus may provide some clues for understanding chronic low dose ionizing radiation-induced cognitive impairment, neurological and neuropsychological disorders.

As some age-associated DNA methylation patterns are similarly observed in both the hippocampus and the peripheral blood cells [199], it may be useful to explore blood biomarkers as indicators of epigenetic changes that occur in the hippocampus, as this may have significant translational relevance.

## Figures and Tables

**Table 1:** Summary of the epigenetic regulation of different cell types in the hippocampus.

Cell Type	DNA Methylation	Histone Modifications	Non-Coding RNAs (ncRNAs)
Principal neurons	- Developmentally regulated by a DMP - Inhibition of DNMTs caused an inability to consolidate memories and induce LTP	- Histone methyltransferase MLL2 (KMT2B) crucial for memory formation - Decreases in H3K4me3 and H3K4me2 correlated with impaired memory function - Reduction in H3K9ac leads to memory impairment - Histone methylation crucial for hippocampal consolidation of memories	- Disruption in miR-137-regulated H3K27me3 levels leads to spatial memory deficits - miR-467b-5p increase contribute to deficits in cognition and behavior
Interneurons	- DNMT1 binding to GAD67 promoter in GABAergic interneurons cause deficits in hippocampal neurogenesis		- lncRNA-Evf2-knockout mice have significantly reduced interneurons
Astrocytes	- DNA methylation regulates Kir4.1 in potassium buffering crucial for protecting neurons	- Significant decrease in H3K9me3 in C9ALS/FTD BAC mouse models - H2AX phosphorylated more in AD patients	- Astrocytic miR-324-5p facilitates the formation of synapses - Astrocytic death post-epilepsy regulated by miR-34b-5p - Increase in miR-132 in epileptogenic hippocampus in rats and humans - miR-191a promotes restoration of CA1 neurons in rat hippocampus
Microglia	- AD may be partly attributed to global DNA methylation changes in microglia - IL1β expression in aging microglia regulated by DNA methylation	- HDAC inhibition promotes neuronal LTP - HDACs 1 and 2 modulate microglial function in the hippocampus in homeostasis, development and neurodegeneration	- miR-301b overexpression accelerated hippocampal microglial activation and cognitive impairment - miR-155 involved in inflammation-induced neurogenic deficits through microglial activation in hippocampus - miR-146a restores learning and memory impairment by suppressing hippocampal microglial inflammation
Oligodendrocytes	- DNMT1 crucial for oligodendrocyte differentiation and proliferation - MS patients have upregulated levels of DNA methyltransferases (DNMT1, DNMT3A and DNMT3B) - Loss of myelin correlates with DNA methylation changes	- Oligodendrocytes with deacetylated histones seen in early MS lesions - Oligodendrocytes with high levels of histone acetylation seen in chronic MS patients - Inhibition of class I HDACs inhibits specification of OPCs from NPCs - HDACs 1, 3 and 10 promote oligodendrocyte differentiation	- miR-23 overexpression enhances oligodendrocyte differentiation and myelin thickness via mTOR/AKT - miR-124 associated with demyelination in hippocampus and memory dysfunction

DNMT: DNA methyltransferase; HDAC: histone deacetylase; NPC: neural progenitor cell; mTOR: mammalian target of rapamycin; AKT: protein kinase B; MS: multiple sclerosis; AD: Alzheimer’s disease; LTP: long-term potentiation; DMP: DNA methylation program; OPCs: oligodendrocyte progenitor cells.
